# Gut microbiota and intestinal immunity interaction in ulcerative colitis and its application in treatment

**DOI:** 10.3389/fcimb.2025.1565082

**Published:** 2025-04-09

**Authors:** Fan Bu, Kaiyuan Chen, Siche Chen, Yi Jiang

**Affiliations:** Department of Colorectal Surgery, Zhejiang Provincial People’s Hospital, Affiliated People’s Hospital of Hangzhou Medical College, HangZhou, China

**Keywords:** gut microbiota, fecal microbiota transplantation, inflammatory diseases, intestinal immunity, herb medicine

## Abstract

Ulcerative colitis (UC) is a chronic, non-specific inflammatory bowel disease characterized by inflammation and injury of the colonic mucosa, exhibiting an increasing global incidence. Although research into UC pathogenesis is ongoing, the precise mechanisms remain to be fully elucidated. Studies indicate that UC development results from a complex interplay of factors, including genetic predisposition, environmental exposures, gut microbial dysbiosis, and immune dysregulation. Specifically, UC pathogenesis involves aberrant immune responses triggered by interactions between the host and gut microbiota. A complex, dynamic relationship exists between the microbial community and the host immune system throughout UC pathogenesis. Accumulating evidence suggests that changes in microbiota composition significantly impact gut immunity. This review will examine the intricate balance between the gut microbiota and mucosal immunity in UC progression and discuss potential therapeutic applications, providing a reference for further clinical treatment of this patient population.

## Introduction

Ulcerative colitis (UC) is a chronic, nonspecific inflammatory bowel disease characterized by continuous inflammation of the colonic mucosa and submucosa. This inflammation typically initiates in the rectum and extends proximally along the colon (Ng et al., 2017). The etiology of UC is complex and involves an interplay between genetic susceptibility, environmental triggers, and dysregulated immune responses to commensal gut microbiota (GM) (Xavier and Podolsky, 2007; McGovern et al., 2010). Current therapeutic strategies include anti-inflammatory agents, immunosuppressants, biologics targeting cytokines or integrins, and surgical intervention for refractory cases (Ardizzone and Bianchi Porro, 2005). However, many patients experience recurrent symptoms, necessitating the exploration of novel therapeutic targets.

Advances in research have established the significant role of the gut microbiota and intestinal mucosal immune system in the pathogenesis of UC. Studies have consistently demonstrated gut dysbiosis in UC patients, with metagenomic analyses revealing a reduction in both the richness and diversity of the gut microbiota in these individuals, characterized by a decrease in beneficial bacteria, notably *Bifidobacterium*, *Lactobacillus*, and *Akkermansia muciniphila* (*A. muciniphila*). Conversely, an increase in pathogenic bacteria, including adherent-invasive *Escherichia coli*, *Clostridium difficile*, and *Mycobacterium avium* subspecies paratuberculosis, has been observed (Humbel et al., 2020).

Gut dysbiosis and increased intestinal epithelial barrier permeability may lead to activation of the intestinal immune system, inducing the production of pro-inflammatory cytokines within local tissues, including interleukin-1β (IL-1β), interleukin-6 (IL-6), tumor necrosis factor-α (TNF-α), and interferon-γ (IFN-γ) (Kobayashi et al., 2020). Furthermore, extra-intestinal inflammatory manifestations are frequently observed in UC patients. For example, peripheral neutrophils exhibit elevated expression of inflammatory mediators, such as S100A8, S100A9, TNF-α, and IL-6 (Pavlidis et al., 2022). The peripheral blood neutrophil-to-lymphocyte ratio (NLR) has been documented as a reliable indicator of stress and inflammatory burden in patients (Su et al., 2022). The underlying pathological mechanisms can be classified into two primary components: firstly, neutrophilia; and secondly, lymphopenia. During inflammatory processes, particularly in the acute phase, the organism releases a spectrum of chemokines and cytokines that recruit neutrophils to the inflammatory site. Neutrophils, integral components of the innate immune system, engage in phagocytosis of pathogens and release enzymes and other mediators to eradicate invading organisms. However, in chronic inflammatory conditions such as UC, persistent neutrophil hyperactivity can contribute to host tissue damage. Conversely, lymphopenia, a decrease in circulating lymphocyte counts, can arise from chronic inflammatory states. Lymphocytes, encompassing T cells and B cells, are pivotal in the regulation of the immune response. Inflammatory diseases may result in heightened lymphocyte apoptosis or their migration to sites of inflammation, resulting in a relative reduction in peripheral lymphocyte numbers (Langley et al., 2021; Su et al., 2022; Dusunceli et al., 2025). This review aims to explore the intricate interplay between the gut microbiota and mucosal immunity in the context of UC, and to evaluate the efficacy and mechanisms of potential therapeutic interventions, including fecal microbiota transplantation (FMT), probiotics, prebiotics, and Traditional Chinese Medicine (TCM). By comprehensively understanding the modulation of the gut microbiota-immune axis by these treatments, we hope to uncover the potential of targeting this axis as a therapeutic strategy for UC. Future research endeavors should continue to explore these areas to enhance our knowledge and develop innovative management strategies for UC.

## Gut microbiota and UC

The advent of advanced sequencing technologies has facilitated the elucidation of the complex microbial community residing within the human gut, encompassing bacteria, fungi, and viruses (Langley et al., 2021). This gut microbiota plays a pivotal role in nutrient absorption, host energy provision, and the maintenance of the intestinal biological barrier. Investigations have revealed that patients with UC demonstrate significant alterations in the abundance and distribution of their gut microbiota, resulting in compromised intestinal mucosal barrier integrity. The proliferation of pathogenic bacteria and their subsequent secretion of enterotoxins contribute to increased gut permeability, dysregulation of gut immunity, and the consequent development of chronic intestinal inflammation. Besides, studies have documented a reduction in the richness and diversity of the gut microbiome in UC patients, characterized by a decrease in beneficial bacteria such as *Bifidobacterium* and *Lactobacillus* sp*ecies*. Conversely, an increase in pathogenic bacteria, including adherent-invasive *Escherichia coli*, *Clostridium difficile*, and *Mycobacterium avium subspecies paratuberculosis*, has been observed (Chassaing and Darfeuille-Michaud, 2011).

Current evidence suggests that mice raised under germ-free conditions do not develop ulcerative colitis, and antibiotic administration can partially alleviate symptoms of colitis (Gordon et al., 2022). Interestingly, a recent study demonstrated that the transfer of gut microbiota from UC patients to germ-free mice could induce colitis, supporting the concept of a close relationship between gut microbiota and inflammation in the etiology of the disease (Du et al., 2015; Cui et al., 2018). Moreover, colonization with a normal human gut microbiota can exert therapeutic effects on UC by enhancing intestinal mucosal barrier function, modulating the production of anti-inflammatory cytokines, and suppressing the proliferation of pathogenic bacteria within the gut. FMT is increasingly utilized in clinical practice as a potential therapeutic intervention for UC, aiming to restore gut microbiota homeostasis through the transfer of fecal material from healthy donors to patients. In a dextran sulfate sodium (DSS)-induced colitis mouse model, FMT was found to increase body weight, colon weight, and colon length while decreasing the expression of key cytokines and the oxidative state in the colon. This restorative effect of FMT is achieved by increasing the relative abundance of *Firmicutes* and decreasing the relative abundance of *Bacteroidetes* and *Proteobacteria (* Zhang et al., 2020). Gut microorganisms can be categorized into three main types based on their primary functions:

1. Probiotics

Probiotics are defined as live microorganisms that, when administered in adequate amounts, confer a health benefit on the host by colonizing the human gastrointestinal and reproductive tracts. They inhibit the growth of pathogenic bacteria through the production of antimicrobial substances, such as short-chain fatty acids and secondary bile acids, thereby improving the host’s microecological balance. Furthermore, probiotics stimulate the host’s non-specific immune function, enhancing the activity of immune cells and thus improving the body’s resistance to disease (Goodoory et al., 2023). In the context of UC, probiotics can modulate the immune system by stimulating the production of secretory immunoglobulins, alleviating inflammation, reducing the permeability of the intestinal mucosa, inhibiting epithelial cell apoptosis, and reinforcing the gut barrier. *Lachnospiraceae* are abundant obligate anaerobes in the human gut that can restore hematopoietic function, repair the gastrointestinal tract, and enhance resistance to colonization by enteric pathogens through the production of butyrate and involvement in bile acid metabolism. *Ruminococcaceae* have been reported as key microbial agents in the conversion of primary to secondary bile acids and the promotion of intestinal stem cell proliferation. *Akkermansia muciniphila* is a prevalent gut commensal bacterium renowned for its capacity to metabolize mucin. By degrading mucin, it promotes the secretion of additional mucus by goblet cells, thereby enhancing the intestinal barrier, which plays a crucial role in maintaining host gut health. Research has demonstrated that *A. muciniphila* can stimulate both innate and adaptive immunity. For instance, studies have indicated that *A. muciniphila* can promote the differentiation of regulatory T cells (Tregs), aiding in the suppression of excessive immune responses. Furthermore, *A. muciniphila* modulates immune responses in the spleen, intestines, and mesenteric lymph nodes through its outer membrane protein Amuc_1100, subsequently ameliorating enteritis symptoms. Recent studies suggest that *A. muciniphila* may contribute to the alleviation of UC symptoms by activating the Nrf2 pathway, thereby promoting macrophage polarization towards an anti-inflammatory phenotype. *Lactobacillus johnsonii* (*L. johnsonii*) is a species within the genus *Lactobacillus*. Studies have shown *L. johnsonii* to be a potentially beneficial bacteria. Studies have shown that *L. johnsonii* colonization is reduced in colitis mice, and oral administration of *L. johnsonii* can alleviate colitis symptoms in these models. Macrophage immune responses are implicated in the anti-inflammatory effects of *L. johnsonii* in colitis. Supplementation with *L. johnsonii* failed to protect against inflammatory infiltration and crypt damage following chemical macrophage depletion. Further investigations have revealed that *L. johnsonii* could activate intestinal CD206+ macrophages, thereby promoting IL-10 secretion and alleviating colitis (Jia et al., 2022). Probiotics are increasingly utilized clinically to improve the condition of UC patients by augmenting probiotic populations and modulating gut microbiota distribution, thereby enhancing microbial homeostasis, suppressing inflammatory responses, and restoring the intestinal barrier. Research indicates that traditional probiotics, including *Bacillus*, *Bifidobacterium*, *Lactobacillus*, and *Saccharomyces cerevisiae*, exhibit varying degrees of efficacy in ameliorating IBD. However, these trials were characterized by relatively small sample sizes. Next-generation probiotics, based on mechanistic research, have identified certain strains of *Faecalibacterium prausnitzii*, *Roseburia* spp., and *A. muciniphila* as promising candidates for improving gut microbiota homeostasis in UC patients (Liu et al., 2021).

A study showed that *Bifidobacterium longum 536* ameliorated clinical symptoms in patients with mild to moderate Crohn’s disease (CD). However, while probiotics may confer benefits under specific circumstances, a comprehensive evaluation of the risks and benefits is warranted prior to initiating treatment in UC patients (Tamaki et al., 2016).

2. Pathogens

Pathogenic microorganisms produce deleterious substances that can compromise host health and induce disease. These pathogens establish infection through adhesion and colonization, followed by invasion, proliferation, and spread. They eventually cause adverse host reactions via toxin production. For instance, lipopolysaccharides (LPS), components of Gram-negative bacterial cell walls, are released upon bacterial lysis. While LPS exhibits relatively low intrinsic toxicity, it can induce systemic inflammatory responses, including fever and shock, during infections (Baumler and Sperandio, 2016). Numerous pathogens, including *Enterobacteriaceae*, *Enterococcus*, and *Escherichia* species, have been implicated in the onset and progression of IBD. Recent investigations comparing the fecal microbiota of UC patients with that of healthy individuals revealed *Enterococcus faecium* as the most differentially abundant species. *E. faecium* strains isolated from the feces of UC patients were shown to promote colitis pathology scores and inflammatory cytokine expression in IL-10 knockout mice, compared to strains isolated from healthy individuals (Seishima et al., 2019). These findings indicate that alterations in the gut microbiome can lead to disease onset in normal mice or exacerbate pre-existing conditions in susceptible mice.

3. Opportunistic pathogens

Primarily composed of Gram-positive bacteria, opportunistic pathogens do not typically induce disease under normal physiological conditions. However, they can breach host defense mechanisms and cause infection under specific circumstances. For instance, in states of compromised host immune function, opportunistic pathogens can more effectively evade immune surveillance and initiate infection. They may modify their surface structures to circumvent recognition by the immune system or secrete substances that inhibit immune cell activity. In healthy individuals, these three categories of gut microbiota engage in mutual antagonism, maintaining a relative equilibrium to ensure the stability of the intestinal environment.

Recent research analyzing colonic mucosal microbiota samples from 28 pairs of UC patients and their healthy spouses identified four potential opportunistic pathogens: *Clostridium tertium*, *Odoribacter* sp*lanchnicus*, *Ruminococcus gnavus*, and *Flavonifractor plautii*. These bacteria, having escaped their suppressed state within the microbiota of UC patients, showed a significant increase in abundance, potentially leading to more inflammatory responses and pathophysiological changes. The increase in these four opportunistic pathogens might be related to the reduction in short-chain fatty acids (SCFAs), which are typically produced by obligate anaerobic bacteria in the gut and contribute to the maintenance of intestinal health. Furthermore, these pathogens may exert influence on the immune system, for instance, by activating mast cells and releasing leukotrienes, thus enhancing IgE responses and amplifying local intestinal inflammation. Therefore, during the onset of UC, the emergence of opportunistic pathogens may result from an imbalance in gut microbiota, and their proliferation could exacerbate inflammation of the intestinal mucosa, further driving disease progression (Li et al., 2021).

## Intestinal immunity and UC

Research indicates that the pathogenesis of UC is closely associated with an imbalance in immune system homeostasis. The pathological process involves excessive activation of the immune response, resulting in immune-mediated damage to the intestinal wall, which also constitutes the pharmacological basis for the effectiveness of corticosteroids, immunosuppressants, and biologics in treating UC (Xavier and Podolsky, 2007; McGovern et al., 2010). The intestinal barrier demarcates the human body from the microorganisms inhabiting the gut, providing essential protection and facilitating interactions between the host and the microbiome. Increasing evidence suggests that disruption of the barrier function may contribute to the initiation and progression of UC. The gut immune system encompasses physical, chemical, and immunological barriers. The physical barrier serves as the first line of defense against external pathogens. It consists of multiple layers that collectively maintain the integrity of the gut and prevent direct entry of pathogens, toxins, and other deleterious substances into the body. Components of the physical barrier include the intestinal epithelial cells, mucus layer, tight junctions, and the gut microbiota.

Intestinal epithelial cells, constituting the innermost layer of the intestine, are primarily composed of a single layer of columnar epithelial cells. These cells are interconnected by tight junctions (TJs), adherens junctions (AJs), and desmosomes, forming a relatively impermeable barrier (Kai, 2021).

Columnar cells, the most prevalent type of intestinal epithelial cells, are responsible for the absorption of water, electrolytes, and nutrients from the digestive contents. Goblet cells produce and secrete mucus, which lubricates the intestinal contents and protects the intestinal wall from mechanical damage and pathogens. Resistin-like molecule beta (RELMβ), a protein produced by goblet cells, functions as a conduit between the gut lumen and the immune system by presenting antigens to dendritic cells (Herbert et al., 2009; McDole et al., 2012). Endocrine cells regulate digestion, nutrient absorption, and energy metabolism through the secretion of diverse hormones and neurotransmitters. Paneth cells, located at the base of the crypts in the small intestine, inhibit the proliferation of bacteria, fungi, and certain viruses by secreting a variety of antimicrobial peptides, such as alpha-defensins and the human-derived HD5 and HD6 (Brubaker, 2023).

The colonic mucosa secretes a substantial quantity of mucus, characterized by complex and extensively O-glycan-modified mucin backbones (Johansson et al., 2013). This mucus layer serves to segregate luminal bacteria from epithelial cells through the following mechanisms (Hansson and Johansson, 2010): (1) Differential densities within the mucus layer establish a compartmentalization, isolating bacteria residing in the outer, less dense layer from those in the inner, denser layer; (2) Continuous mucus secretion facilitates bacterial entrapment and subsequent elimination via the intestinal tract; (3) The primary physiological role of mucus is to function as a lubricant within the gut lumen, thereby aiding in fecal expulsion and mitigating epithelial abrasion.

As the primary mucus-secreting cells, goblet cells are significantly reduced in the intestinal epithelium of UC patients, leading to insufficient synthesis and secretion of MUC2. Furthermore, the levels of goblet cell differentiation factors HATH1 and KLF4 are markedly reduced in UC patients (Gersemann et al., 2009). CD, while sharing classification under IBD with UC, is characterized by thickened mucus layers and increased mucus production. Despite this, the intestinal epithelial barrier in CD patients fails to prevent bacterial invasion. This highlights that the structure and function of mucins, in addition to their quantity, are critically important in IBD (Johansson et al., 2014; Sun et al., 2016). A study observed that mice deficient in Core 1 glycosyltransferase exhibited MUC2 mucins with shortened O-glycans and accelerated degradation, subsequently developing severe colitis (Bergstrom et al., 2017). Another study revealed a thickened mucus layer in CD, suggesting an increase in MUC2 expression and goblet cell hyperplasia; however, the structure of MUC2 is altered due to a 50% reduction in oligosaccharide chain length, leading to a loss of mucus viscoelastic properties and, consequently, a loss of protective function (Fu et al., 2011). Research indicates an increase in sulfate-reducing bacteria and a significant elevation in sulfide metabolism in UC patients and DSS-induced UC mouse models. The presence of sulfide disrupts disulfide bonds between mucins, dismantling the mucus layer network within the gut and increasing the susceptibility to bacterial invasion (Ijssennagger et al., 2015; Ijssennagger et al., 2016). Murine disease models have further confirmed the role of the mucus barrier and mucins in the prevention and development of IBD. In this respect, it was reported that *MUC2* gene-knockout animals developed spontaneous colitis five weeks postnatally and exhibited increased susceptibility to DSS-induced colitis, likely due to the absence of MUC2, which impairs the ability to prevent gut microbiota invasion, resulting in direct contact with epithelial cells and the initiation of colitis (Van der Sluis et al., 2006).

TJs are specialized, multifunctional adhesive proteins that exist between cells, occluding the intercellular space, regulating ion flux within tissues, and maintaining cellular polarity. TJs are located at the boundary of the apical and lateral membranes of adjacent epithelial cells in the colon. At the molecular level, TJs are highly diverse structures comprising transmembrane proteins and cytoplasmic proteins (Tang, 2006). Proteins of the claudin family are essential components of TJs. Besides claudins, TJs contain three other classes of transmembrane proteins: occludin, tricellulin, and junctional adhesion molecules (JAMs). Studies have demonstrated that TJ structures are aberrant in UC patients (Hering et al., 2012). Genetic analysis has identified hepatocyte nuclear factor 4 alpha (HNF4α) as a gene associated with TJ function, which is closely implicated in the pathogenesis of IBD (Ahn et al., 2008). HNF4α is a transcription factor involved in the migration and maturation of colonic cells from the crypts and regulates the expression of claudins, including claudin 2, claudin 5, and claudin 7, whose expression is closely related to barrier function and IBD development. One study revealed that mice deficient in claudin 7 developed fatal colitis shortly after birth (Tanaka et al., 2015). Despite near-identical TJ structures in wild-type and claudin 7 knockout animals, the properties and functions of TJs are compromised in the knockout mice. The epithelial barrier in claudin 7 knockout animals exhibits increased permeability to small molecules. Therefore, claudin 7 may regulate the permeability of small molecules, and its dysfunction can increase gut permeability, promoting the passage of luminal antigens through the epithelial barrier, leading to “leaky gut”, triggering inflammation, and causing colitis (Tanaka et al., 2015).

In addition to physical barriers, such as the mucus layer and tight junctions, various immune cells within the gut collectively maintain intestinal homeostasis. Among these, T cells, B cells, macrophages, dendritic cells, neutrophils, and natural killer (NK) cells play crucial roles in the pathogenesis of UC. Gut T cells are essential in maintaining intestinal immune homeostasis and responding to pathogen invasion. Two T helper cell subsets, Th1 and Th17, are particularly important in the development of UC. Th1 cells primarily secrete IFN-γ and TNF-α, whereas Th17 cells mainly produce IL-17. The excessive activation of Th17 cells is considered a key driver of chronic inflammation in UC (Zhao et al., 2023).

Tregs, possessing immunosuppressive functions, can attenuate excessive immune responses through the secretion of anti-inflammatory cytokines, such as IL-10. In patients with UC, Treg function may be compromised, resulting in an inability to effectively suppress the activities of Th1 and Th17 cells, thereby exacerbating inflammation (Campbell et al., 2018). During the progression of UC, the differentiation of pro-inflammatory Th17 cells is abnormally active, while Tregs that inhibit Th17 activity are diminished. The overproduction of inflammatory cytokines, such as IL-17, by Th17 cells surpasses the immune tolerance conferred by Tregs, leading to tissue damage and the induction of UC (Yan et al., 2023).

B cells not only produce antibodies but also contribute to the inflammatory process through cytokine secretion. In patients with UC, autoantibodies produced by B cells may target normal intestinal tissues, thereby exacerbating inflammation. Furthermore, B cells can function as antigen-presenting cells (APCs), presenting pathogen-derived antigens to T cells, leading to the overactivation of T cells, particularly Th1 and Th17 cells, which results in chronic inflammatory responses (Smillie et al., 2019). A recent study has found that during intestinal mucosal repair, a substantial accumulation of B cells occurs at sites of injury. Notably, B cells were found to partially impede the interaction between epithelial and stromal cells, exerting a degree of negative influence on the intestinal injury repair process (Uzzan et al., 2022). One study showed that IFN-induced B cells (highly expressing Ly6a, Cd274) enriched in damaged areas, up-regulated genes such as Zbp1, exacerbated inflammation and inhibited reparation-related pathways. *In vitro* co-culture experiments, activated B cells significantly reduced the survival rate of epithelial organoids, and IFN stimulation had a more significant effect. At the same time, after B cell clearance, the interaction between stromal cells and epithelial cells is enhanced, extracellular matrix remodeling and growth factor signaling pathways (such as Hippo, PI3K-Akt) are activated (Frede et al., 2022).

Macrophages are a crucial component of the innate immune system, playing roles such as pathogen phagocytosis, clearance of apoptotic cells, and modulation of immune responses. Based on their activation status and functional characteristics, macrophages can be broadly classified into two primary phenotypes: M1 macrophages (classically activated) and M2 macrophages (alternatively activated). During the initial phases of UC, M1 macrophages contribute to the amplification of inflammatory responses and exacerbate tissue damage through the release of pro-inflammatory mediators, including TNF-α, IL-1β, and IL-6. These pro-inflammatory mediators recruit additional immune cells to the inflammatory site, establishing a positive feedback loop that further intensifies the inflammatory cascade (Zhang et al., 2022). In the chronic or resolution phases of UC, M2 macrophages facilitate the attenuation of inflammation and promote the repair of damaged tissues by secreting anti-inflammatory cytokines, such as transforming growth factor beta (TGF-β) and IL-10, as well as participating in tissue remodeling and angiogenesis. However, under certain pathological conditions, excessive M2 macrophage activation may contribute to fibrosis, thereby compromising intestinal function. The adipocyte-dependent microenvironment within the creeping fat of patients with CD has been reported to promote an M2 macrophage subtype with secretion of large amounts of profibrotic factors such as TGF-β, leading to intestinal fibrosis (Kredel et al., 2013).

Dysregulation of intestinal M1/M2 macrophage polarization is closely associated with intestinal inflammatory disease in UC patients. Therefore, restoring the balance of M1/M2 macrophage polarization represents a potentially valuable therapeutic strategy for UC (Yang et al., 2022). It is widely thought that anti-TNF-α therapy may target intestinal macrophages, leading to macrophage depletion and a shift in macrophage phenotype from M1 to M2 (Nazareth et al., 2014).

Dendritic cells, similar to macrophages, play a critical role in homeostasis and the development of IBD through the phagocytosis of cellular debris, cytokine production, tissue repair regulation, and interactions with other cell types. Dendritic cells serve as a critical link between the innate and adaptive immune systems by presenting antigens to and activating T cells. They exhibit a propensity to accumulate at specific locations within the gut, such as Peyer’s patches, isolated lymphoid follicles, and gut-associated lymphoid tissue (De Schepper et al., 2018). A study revealed that dendritic cells isolated from the mucosa of UC patients demonstrated elevated expression of CD40 and increased secretion of IL-6 and IL-12 compared to those from healthy individuals (Al-Hassi et al., 2014).

Neutrophils constitute the most abundant population of immune cells in human circulation and represent the predominant immune cell type within the colonic tissue of UC patients (Kobayashi et al., 2020). The extensive recruitment of neutrophils to the colon and their subsequent activation lead to the formation of neutrophil extracellular traps (NETs), which play a significant role in UC pathogenesis (Dinallo et al., 2019). Studies have found that NETs are markedly elevated in the serum and colonic mucosal tissues of UC patients, capable of inducing inflammatory responses and exacerbating the severity of UC (Li et al., 2020). Neutrophils contribute to the pathogenesis of UC through various mechanisms, including impairment of epithelial barrier function, tissue destruction via oxidative and proteolytic injury, and maintenance of inflammation through the release of multiple inflammatory mediators (Brazil et al., 2013; Brazil and Parkos, 2016). Extensive evidence indicates that the widespread infiltration of neutrophils within the intestinal mucosa is a hallmark of tissue damage in UC, correlating with endoscopic severity and systemic markers of inflammation. NETosis, a form of programmed cell death specific to neutrophils, is characterized by the release of NETs. NETs are web-like structures composed of decondensed chromatin fibers decorated with antimicrobial proteins and granular enzymes, such as neutrophil elastase, myeloperoxidase, and cathepsin G (Delgado-Rizo et al., 2017). This unique mechanism enables neutrophils to trap and eliminate pathogens, including bacteria, fungi, and certain viruses, extracellularly, thereby preventing the dissemination of infection. The process of NETosis involves several steps (Brinkmann and Zychlinsky, 2012):Activation: Neutrophils are activated by various stimul. Chromatin Decondensation: Upon activation, the nuclear envelope breaks down, and the chromatin undergoes decondensation. This is facilitated by the enzymatic activity of peptidylarginine deiminase 4 (PAD4), which citrullinates histones, leading to chromatin decondensation. Membrane Permeabilization: The plasma membrane becomes permeable, allowing the mixing of nuclear and cytoplasmic contents. Release of NETs: The neutrophil releases its nuclear material and granule proteins into the extracellular space, forming NETs. While NETosis is a crucial defense mechanism against pathogens, excessive or dysregulated NETosis can contribute to various pathological conditions (Hakkim et al., 2011).

It has been established that NK cells constitute a population of CD3−CD56+ innate immune cells (Trinchieri, 1989). The majority of studies have focused on NK cells isolated from peripheral blood mononuclear cells (PBMCs). NK cells are involved in both innate and adaptive immunity, capable of recognizing molecules on the cell surface induced by stress signals and viral infections. In the gut, NK cells are dispersed within the epithelium or stroma, where they come into contact with a wide array of antigens, not only components of the microbiota but also those from commensal or pathogenic microorganisms. Intestinal NK cells interact with epithelial cells, fibroblasts, macrophages, dendritic cells, and T lymphocytes, contributing to the maintenance of immune homeostasis and the development of effective immune responses (Tatiya-Aphiradee et al., 2018). NK cells play a crucial role in the response to bacterial infections in the gut, primarily through the production of IFNγ, which can stimulate the recruitment of additional NK cells from the peripheral blood, leading to an amplified antibacterial immune response (Wang et al., 2017). In active UC, peripheral NK cell levels are significantly reduced compared to those in inactive UC. Following anti-TNF therapy, responsive patients exhibit significantly elevated peripheral NK cell counts compared to non-responsive UC patients. In UC, NK cells can be targeted by 6-mercaptopurine, a medication that induces apoptosis and NK cell depletion, potentially attenuating the inflammatory response (Yusung et al., 2017).

In summary, the disruption of the gut barrier in UC involves multiple levels, including the loss of physical barrier integrity, attenuation of chemical barrier function, dysregulation of the immune system, and dysbiosis of microbial communities. These factors synergistically contribute to the pathological progression of UC.

## Interaction between gut microbiota and intestinal mucosal immunity in UC

The interaction between the gut microbiota and the host immune system commences at birth, with the microbiota influencing the development of the immune system, which, in turn, modulates the composition of the microbiota. This bidirectional relationship propagates through a network of signaling pathways that extend beyond the immune system. These immune-mediated signaling processes, coupled with direct interactions between the microbiota and the host, exert influence on multiple organs such as the gut, liver, muscle, and brain (Rooks and Garrett, 2016). Microbial components and their metabolites play a pivotal role in the regulation of intestinal immunity. Pattern recognition receptors (PRRs) constitute a critical component of the innate immune defense, serving as the first line of defense following pathological injury. They are expressed on a diverse array of immune cells (leukocytes, macrophages, etc.), as well as non-immune cells (epithelial cells, endothelial cells, and neurons), and respond to a broad spectrum of bacterial and viral ligands. PRRs are critical in maintaining homeostatic interactions between the gut and the symbiotic microbiota, possessing the capacity to discriminate between pathogenic and symbiotic organisms (Bersch et al., 2021). PRRs comprise four primary subfamilies: Toll-like receptors (TLRs), nucleotide-binding oligomerization domain-like receptors (NLRs), retinoic acid-inducible gene I-like receptors (RLRs), and C-type lectin receptors (Walsh et al., 2013). Among these, TLRs and NLRs interact with the intestinal microbiota, playing a significant role in the initiation and progression of UC.

TLRs constitute a family of functional type I transmembrane glycoproteins widely expressed in epithelial cells. They recognize microbial pathogen-associated molecular patterns (PAMPs) and host-derived damage-associated molecular patterns (DAMPs), thereby initiating inflammatory responses (Keogh et al., 2021). TLR1, TLR2, TLR4, TLR5, and TLR6 are expressed in dendritic cells (DCs), neutrophils, and macrophages, where they identify microbial membrane components. In contrast, TLR3, TLR7, TLR8, and TLR9 are expressed in intracellular vesicles, such as endosomes, playing a role in the recognition of microbial nucleic acids (Qing et al., 2022). The majority of TLRs signal through the adapter protein MyD88. However, TLR4 utilizes both MyD88-dependent and MyD88-independent pathways. Activation of TLRs via MyD88 induces NF-κB, mitogen-activated protein kinase (MAPK), and AP-1 signaling cascades, while the MyD88-independent pathway leads to the stimulation of interferon regulatory factor 3/7 (IRF-3/-7) signaling. The gut microbiota mediates host immune responses by activating TLR pathways in the intestine. Through TLR/MyD88 signaling, the intestinal microbiota restricts the trafficking of CX3CR1 high phagocytes from the lamina propria to mesenteric lymph nodes (MLNs) (Diehl et al., 2013). Gut microbiota dysbiosis results in CCR7-dependent migration of CX3CR1 high phagocytes to MLNs, leading to enhanced Th1 responses against non-invasive pathogens and increased IgA production in MLNs (Ueda et al., 2010). Studies have shown that *Clostridium butyricum*, a probiotic, promotes the production of the protective anti-inflammatory cytokine IL-10 by CD11b+ CD11c intermediate F4/80+ intestinal macrophages through the Toll-like receptor 2 (TLR2)/MyD88 signaling pathway, thereby ameliorating symptoms of DSS-induced colitis in mice (Hayashi et al., 2013). The TLR2 pathway is also closely associated with the effects mediated by *Bacteroides fragilis (B. fragilis*) *(* An et al., 2014). *B. fragilis* inhibits invariant natural killer T (iNKT) cell proliferation and alleviates colitis through the production of glycosphingolipids and capsular polysaccharide A (Mazmanian et al., 2008; Dasgupta et al., 2014). Non-toxigenic *Bacteroides fragilis* (NTBF) promotes intestinal immune tolerance by engaging TLR2 on dendritic cells via its polysaccharide A (PSA). Similarly, NTBF outer membrane vesicles (OMVs), which are rich in PSA, activate TLR2-mediated IL-10 production, enhancing the generation of Tregs to prevent experimental colitis (Shen et al., 2012; Chan et al., 2019). A recent study, conducting microbiota analysis on 20 healthy individuals and 46 patients with UC, revealed a significant reduction in *A. muciniphila* in UC patients, suggesting this bacterium may serve as a potential probiotic (Shen et al., 2012). *A. muciniphila* may interact with Toll-like receptor 2 through its outer membrane protein Amuc_1100, thereby improving gut permeability (Wang et al., 2020). Recent research has demonstrated that *A. muciniphila* can ameliorate DSS-induced colitis in mice by inducing IgG production and antigen-specific T cell responses, further confirming its role in regulating immune homeostasis (Ansaldo et al., 2019). *Lactobacillus rhamnosus GG* (LGG) has shown clinical benefits for UC patients. A study indicated that LGG extracellular vesicles (EVs) can alleviate colonic tissue damage and shorten colon length (p < 0.01), mitigating intestinal inflammation by inhibiting the activation of the TLR4-NF-κB-NLRP3 axis (Tong et al., 2021).

The nucleotide -binding oligomerization domain-like receptor (NLR) family can be divided into three distinct subgroups: (1) NLRs involved in inflammasome formation (e.g., NLRP1, NLRP3), (2) positive regulatory NLRs (e.g., Nod1, Nod2), and (3) negative regulatory NLRs (e.g., NLRX1, NLRC3). Each subgroup exhibits independent and distinct signaling pathways and downstream effects. Among the NLRP family, NLRP3 is a prototypical member that recruits the adaptor protein ASC and pro-caspase-1 to form a multiprotein complex known as the inflammasome. This process triggers the processing and release of IL-1β and IL-18, playing a pivotal role in regulating gut homeostasis. A study has shown that gut microbiota dysbiosis and a reduction in short-chain fatty acids (SCFAs) are associated with NLRP3 inflammasome activation, leading to impaired intestinal barrier function and exacerbation of colitis (Feng et al., 2019). Nod1 and Nod2 play key roles in the response to specific bacterial pathogens. For instance, enteric pathogenic Citrobacter rodentium induces IL-17 responses through Nod1- and Nod2-dependent pathways (Rubino et al., 2013). The NOD2 receptor is a classical pattern-recognition receptor that senses specific microbial peptidoglycan fragments, including muramyl dipeptide (MDP) and N-acetylglucosamine (NAG)-MDP. Loss of NOD2 function may lead to a lack of inhibition of TLR2 stimulation, resulting in the activation of inflammatory pathways and an excessive Th1 response (Watanabe et al., 2004). Furthermore, the NOD2 3020insC variant has been shown to suppress IL-10 expression in human monocytes, suggesting that NOD2 mutations may result in inadequate immune regulation, indicating that NOD2 is a critical immune receptor in inflammation (Henckaerts and Vermeire, 2007; Noguchi et al., 2009). Studies have demonstrated that the Firmicutes peptidoglycan remodeling enzyme DL-endopeptidase increases the levels of NOD2 ligands in the gut and influences the outcome of colitis (Gao et al., 2022).

In addition to bacteria themselves, bacterial metabolites play a crucial role in modulating immune cells. *Clostridium* clusters VI and XIVa within the gut produce SCFAs through the fermentation of dietary components. SCFAs, including acetate, propionate, and butyrate, modulate gut immunity and suppress intestinal inflammatory responses (Furusawa et al., 2013). Propionate, among SCFAs, promotes its inhibitory activity by suppressing histone deacetylases (HDACs) via the GPR43 signaling pathway, thereby preventing the occurrence of T cell-induced colitis. A significant negative correlation exists between butyrate uptake/oxidation and the Mayo endoscopic subscore, as well as the Geboes histological score (De Preter et al., 2012). In intestinal epithelial cell (IEC) models, butyrate inhibits LPS-induced NF-κB activation through GPR109A, both *in vitro* in colon cell lines and *ex vivo* in mouse colon (Thangaraju et al., 2009). Butyrate increases IL-10 production by human Tregs differentiated *in vitro*, further augmenting the suppressive capacity of human Tregs (Zuo et al., 2022). Bile acids (BAs) regulate immune responses in the intestine and liver through their receptors. Dysregulation of gut BAs is considered a key factor in the pathogenesis of inflammatory bowel disease. The interaction of BAs with TGR5 reduces NLRP3 inflammasome activation and inhibits NF-κB signaling, thus suppressing pro-inflammatory cytokine production (Pols et al., 2011; Biagioli et al., 2017). Beyond TGR5, the bile acid-farnesoid X receptor (FXR) axis in macrophages also inhibits the expression of IL-6, TNF-α, IL-1β, and iNOS through chromatin modifications mediated by nuclear receptor corepressor proteins (Biagioli et al., 2017). A study showed that ursodeoxycholic acid (UDCA), derived from gut microbiota, reduces inflammatory cytokine production by acting on FXR and simultaneously inhibiting NF-κB activation in macrophages (Pi et al., 2023). The types and quantities of BA derivatives produced by gut bacteria vary. Many gut bacteria contain genes encoding bile salt hydrolase (BSH), which are involved in the BA deconjugation process. Due to differences in regulation and substrate specificity, the primary and secondary BA metabolic profiles of these bacteria may be affected. Primary BAs in the gut, along with certain potent secondary BAs such as lithocholic/3-oxo-lithocholic acid, regulate retinoic acid receptor-related orphan receptor gamma (RORγ)+ Tregs through the bile acid receptor vitamin D receptor (VDR) (Song et al., 2020). Human VDR gene variants associated with inflammatory bowel disease may influence disease susceptibility by improperly regulating the pool of gut Tregs. Tryptophan, an essential amino acid, undergoes metabolism via three distinct pathways within the gut: the indole pathway mediated by the gut microbiota, the serotonin system within enterochromaffin cells, and the kynurenine pathway in immune cells and the intestinal mucosa. Specific gut microbial taxa, including *Peptostreptococcus*, *Lactobacillus*, *Escherichia coli*, and species of *Clostridium*, are associated with the production of indole metabolites (Hubbard et al., 2015). Indole metabolites and kynurenines interact with the aryl hydrocarbon receptor (AHR), inducing regulatory T cell differentiation, attenuating Th17 and Th1 responses, and generating anti-inflammatory mediators. AHR is a transcription factor whose activation leads to the production of interleukin-22 (IL-22), which ultimately contributes to mucosal healing, the development of Foxp3+ regulatory T cells, and the reduction of inflammation (Lee et al., 2011; Zelante et al., 2013; Gutierrez-Vazquez and Quintana, 2018). Indole metabolites can alleviate colitis; for example, supplementation with indole metabolites such as indole-3-carbinol (I3C), inhibition of kynurenine monooxygenase (KMO), and selective stimulation or inhibition of specific serotonin receptors can mitigate colitis (Tashita et al., 2020). Indolepropionic acid alleviates DSS-induced colitis in mice, reducing tissue abundance of Th1 cells, increasing the population of regulatory T cells, and enhancing IL-10 production (Aoki et al., 2018). An AHR antagonist reverses the beneficial effects of indolepropionic acid on colitis (Aoki et al., 2018). Indole metabolites also potently inhibit myeloperoxidase (MPO) at physiological concentrations, contributing to the limitation of oxidative stress and tissue damage (Alexeev et al., 2021).

Polyamines (PAs) are small polycationic molecules derived from L-arginine metabolism (Lamas et al., 2016), including putrescine, spermidine, and spermine (SPMD). SPMD regulates the differentiation of CD4+ T cells *in vitro*, preferentially converting naïve T cells into a regulatory phenotype. In a T cell transfer colitis model, dietary supplementation with SPMD promotes the homeostatic differentiation of Tregs within the intestine and reduces pathology. The beneficial effects of SPMD or L-arginine on gut immunity are mediated through the promotion of Treg development. Recently, PAs have been recognized as a class of metabolites with anti-inflammatory properties (Matsumoto et al., 2011; Matsumoto et al., 2012; Kibe et al., 2014), promoting M2 macrophage polarization and the inhibitory activity of tolerogenic DCs (Yang et al., 2016; Mondanelli et al., 2017). Previous studies have reported the beneficial role of L-arginine in regulating gut homeostasis during inflammation and pathological processes (Marc and Wu, 2009; Fritz, 2013). It is proposed that the anti-inflammatory properties of L-arginine may also be attributed to an accompanying increase in PA metabolism. A study demonstrated that SPMD induces Foxp3 expression under Th17 and Treg induction conditions *in vitro*. Moreover, administration of SPMD fosters a tolerogenic environment within the gut and improves inflammation-induced tissue pathology in an adoptive T cell transfer colitis model (Carriche et al., 2021) In germ-free mice, colonization with wild-type *Escherichia coli* (but not polyamine biosynthesis-deficient *E. coli*) increased intracellular polyamine levels in colonic cells, accelerating epithelial renewal. Symbiotic bacteria-derived putrescine augments the abundance of anti-inflammatory macrophages in the colon. Bacterial polyamines improve symptoms of DSS-induced colitis in mice (Nakamura et al., 2021).

Changes in gut microbiota metabolites have also been noted in UC patients. Accumulating evidence suggest that the level of SCFAs in IBD patient’s feces is decreased in varying degrees. For example, Julian R. Marches et al. found that acetic acid and butyric acid in feces of CD and UC patients were lower than those in the healthy group by performing 1H NMR spectroscopy. Another cohort study revealed reduced level of propionic acid and acetic acid by carrying out GC–MS and low abundance of butyrate-producing bacteria in UC patient’s feces (Marchesi et al., 2007; Machiels et al., 2014). Henri Duboc et al. found that SBAs in serum and feces of patients with IBD were reduced, and sulfated LCA lost its anti-inflammatory effect (Duboc et al., 2013).In a clinical study, metabolomics and metagenomics approach have been used to analyze fecal samples from IBD patients and healthy people. The GC–MS analysis showed that the level of putrescine and cadaverine were significantly higher in CD and UC patients (Santoru et al., 2017).

**Figure 1:**
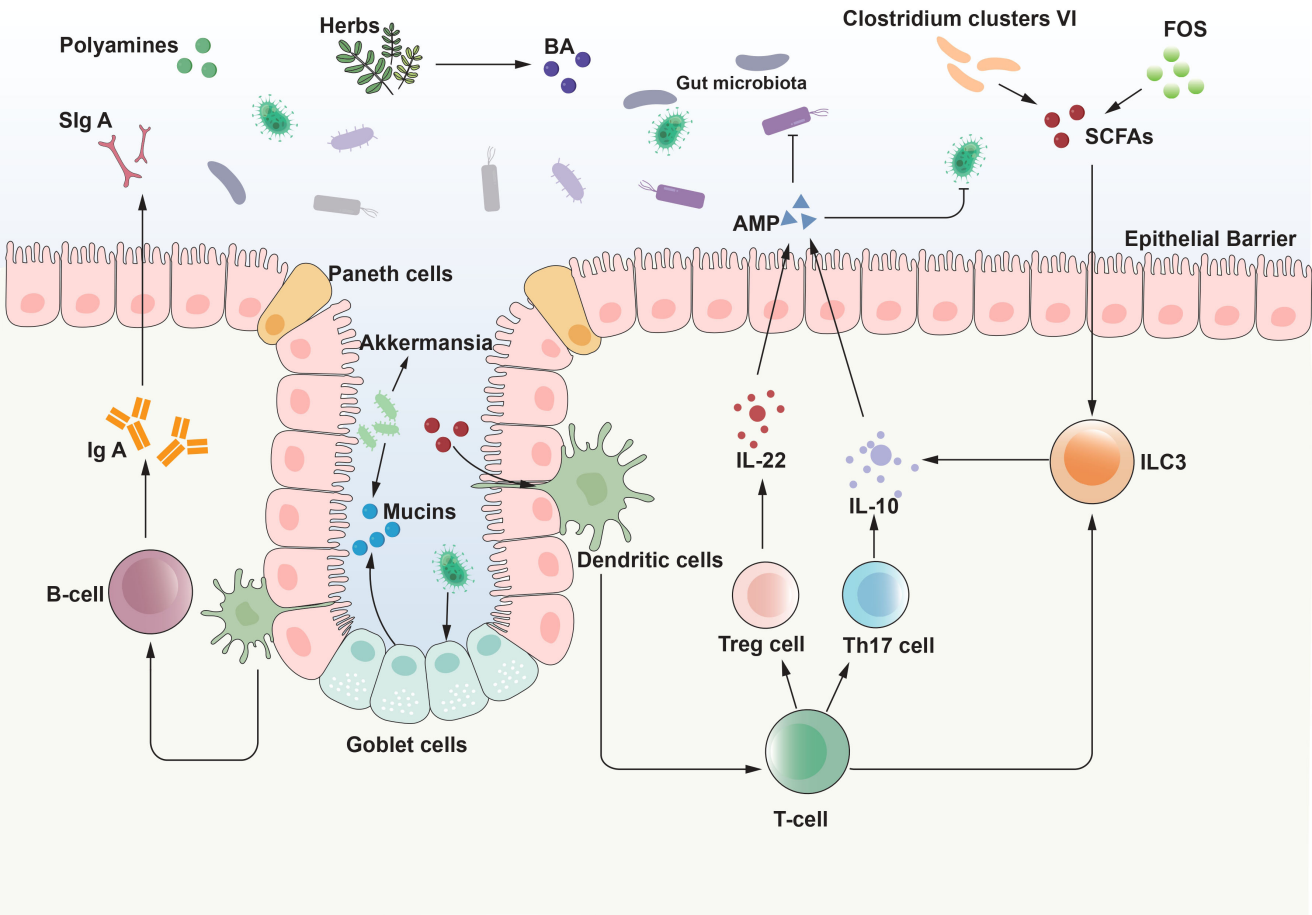
Interaction between gut microbiota and intestinal mucosal immunity. The interaction between gut microbiota and the intestinal mucosal system is the basis for achieving homeostasis of immune function and balance of intestinal ecology. The interaction between the gut microbiota and the host immune system commences at birth, with the microbiota influencing the development of the immune system, which, in turn, modulates the composition of the microbiota. This bidirectional relationship propagates through a network of signaling pathways that extend beyond the immune system. Through the interaction with various intestinal cells, gut microbiota and its metabolites initiate signal transduction pathways, regulate body responses, drive the maturation of intestinal immunity, and then maintain immune balance. AMP, antimicrobial peptide; BA, bile acid; IL, interleukin; ILC3, Type 3 innate lymphoid cells; SCFAs, short-chain fatty acids; SIgA, secretory immunoglobulin A; FOS, fructooligosaccharides. Tregs, regulatory T cells.

In summary, immune cells interact intimately with gut microbiota and their metabolites, collectively maintaining intestinal homeostasis and playing a pivotal role in the pathogenesis and progression of UC (**Figure 1**).

## Application of intestinal immunity and gut microbiota research in the treatment of UC

Patients with UC of varying severity have access to multiple therapeutic options; however, a definitive treatment protocol remains elusive. The therapeutic objectives encompass both short-term and long-term benefits for the patient. For patients with mild to moderate disease, 5-aminosalicylic acid (5-ASA) or low-bioavailability corticosteroids are predominantly employed to achieve effective disease remission (Bonovas et al., 2019). In cases of moderate to severe UC, systemic corticosteroids are typically the initial therapeutic intervention; however, they are not suitable for the safe and effective maintenance of remission. In steroid-dependent patients, thiopurine drugs are considered steroid-sparing agents, although their efficacy remains limited (Gordon et al., 2024).

The role of gut microbiota and intestinal immune interactions in the pathogenesis of UC is irrefutable, offering innovative avenues for UC therapy. The subsequent discussion will examine the application of gut microbiota and intestinal immune interaction in the treatment of UC through methods such as FMT, probiotics, prebiotics, and herbal therapies (**Table 1**).

**Table 1:** Application of intestinal immunity and gut microbiota research in the treatment of UC.

Treatment approach	Model	Beneficial changes achieved	Gut microbiota and its metabolites	Intervention mechanism
Lactobacillus reuteri	C. rodentium infection in mice	ameliorated intestinal inflammation, maintain intestinal epithelial regeneration and repair intestinal damage	reduced C. rodentium colonization	activation of the Wnt/β-catenin pathway, induced differentiation toward Paneth cells, increased antimicrobial peptide expression
Lactobacillus entericus	Dextran Sulfate Sodium (DSS)-induced colitis in mice	protected gut mucosal barrier	Not stated	reduced Th17 cell response
A. muciniphila	DSS -induced colitis mice	recover the loss of body weight, relieved colon shortening	Not stated	Reduced infiltration of cytotoxic T lymphocytes and macrophages in the spleen and mesenteric lymph nodes
Lactobacillus johnsonii	DSS -induced colitis mice	relieve chronic colitis and maintain the colonic mucosal barrier. reduce the degree of intestinal inflammation and subsequent intestinal shrinkage	increased the B/F ratio, The relative abundance of Bacteroidota was enriched whereas Firmicutes and Actinobacteriota were decreased. increased the levels of Prevotellaceae. decreased the levels of Lachnospiraceae, Oscillospiraceae and Erysipelotrichaceae	activating intestinal CD206+ macrophages and promoting their secretion of IL-10
B. longum 536	patients with active ulcerative colitis(UC)	decrease of UCDAI scores, decrease in the Rachmilewitz endoscopic index (EI) and the Mayo sub-score	Not stated	Not stated
Streptococcus faecalis T-110, Clostridium butyricum TO-A, and Bacillus mesentericus TO-A	outpatients with UC in remission	decrease of UCDAI scores	Not stated	Not stated
VSL#3	patients with active UC	a combined induction of remission/response rate of 77%	Not stated	Not stated
fructooligosaccharides (FOS)	Patients with active UC	dyspeptic symptoms scale decreased; reduction in fecal calprotectin	Not stated	Not stated
β-fructans	Patients with active UC	decreased the Mayo score and fecal calprotectin	increased Bifidobacteriaceae and Lachnospiraceae, induction of butyrate and acetate production	reduce colonic inflammation possibly through the stimulation of butyrate-producing bacteria and their activity,
Total flavone of Abelmoschus manihot	DSS -induced colitis mice	improved intestinal barrier integrity	increased the relative abundance of A. muciniphila, Gordonibacter and Erysipelatoclostridium	regulate the NF−kappaB and MAPK signaling pathways
Astragalus membranaceus (Huangqi) extract	DSS -induced colitis mice	alleviated symptoms and histopathological damage	increased the relative abundances of Mur ibaculaceae, Lachnospiraceae, Rikenellaceae and Ruminococcaceae	regulated the balance of Th17/Treg cells and the levels of cytokines
Codonopsis pilosula	DSS -induced colitis mice	decrease of DAI scores, recover the loss of body weight	increased the relative abundances of Bifdobacterium spp., Lactobacillus spp., and Akkermansia spp., and decreased the levels of Desulfovibrio spp., Alistipes spp., and Helicobacter spp. enhanced the production of short-chain fatty acids	restored the T17/Treg balance, inhibited the expression of IL-17A, IL-17F, IL-6, IL-22, and TNF-α
Chlorogenic acid	DSS -induced colitis mice	attenuated weight loss, decreased disease activity index, and improved mucosal damage	decreased the proportion of Firmicutes and Bacteroidetes. enhanced the fecal microbiota diversity, increased the proportions of akkermansia	ameliorated the infiltration of T cells, neutrophils and macrophages
Celastrol	DSS -induced colitis mice	recovered body weight and colon length as well as the decreased disease activity index (DAI) score and intestinal permeability	increased the relative abundances of Prevotellaceae, Alloprevotella, Paraprevotella and Butyricicoccus	improved the balances of Treg/Th1 and Treg/Th17
Baitouweng Tang	DSS -induced colitis mice	reduced the symptoms and histopathological score	the proportion of Firmicutes to Bacteroidetes was decreased, and the ratio of Proteobacteria was decreased. the relative abundance of Escherichia-Shigella was decreased, the relative abundance of Lactobacillus and Akkermansia were increased	Activation of the IL-6/STAT3 pathway
Baitouweng Tang	DSS -induced colitis mice	improved the clinical symptoms such as and histological injury and colon shortening	increased the relative abundance of Firmicutes, Proteo-bacteria, Actinobacteria, Tenericutes, and TM7,decreased the concentrations of UDCA, HDCA, αMCA, βMCA, CA, and GLCA	improved the expressions of the FXR and TGR5
Baitouweng Tang	DSS -induced colitis mice	reduced intestinal permeability of UC mice, increased expression of tight junction proteins	increased the levels of acetate, propionate, isobutyric acid, and isovalerate	regulated the balance between T helper (Th)17 and regulatory T (Treg) cells, decreased interleukin (IL)-1β, IL-6, and tumor necrosis factor-α, and increased IL-10 levels
*Pulsatillae radix* extract	DSS -induced colitis mice	improved the clinical symptom, prevented the shorten of colon length, and decreased the diseased activity index (DAI)	increase in the relative abundance of Bacteroidetes, Deferribacteres, and Proteobacteria phyla and decrease in Firmicutes, decrease in the genera levels of *Bacteroides*, *Parabacteroides*, *Prevotella*, *Mucispirillum*, *Coprococcus*, *Oscillospira*, and *Escherichia*.	up-regulate NOD-like receptor signaling pathway, down-regulate Cytokine-cytokine receptor interaction, and TNF and IL-17 signaling pathways.
Kuijieyuan Decoction (KD)	DSS -induced colitis mice	reduced DAI Scores and increased colon length, improved intestinal barrier injury	increased the proportion of Alloprevotella, Treponema, Prevotellaceae, and Prevotella, and reduced the proportion of Escherichia_Shigella and Desulfovibrio in gut microbiota	affecting TLR4-dependent PI3K/AKT/NF-kB signaling
Codonopsis pilosula Saponins	DSS -induced colitis mice	recovered body weight and colon length as well as the decreased DAI score	stimulating the growth of three important probiotics, i.e., Bifdobacterium spp., Lactobacillus spp., and Akkermansia spp., and inhibiting the growth of pathogenic bacteria, including Desulfovibrio spp., Alistipes spp., and Helicobacter spp. enhanced the production of short-chain fatty acids(acetic acid, propionic acid, butyric acid, isobutyric acid, and isovaleric acid)	upregulated the expression of anti-inflammatory cytokines and downregulated the secretion of pro-inflammatory cytokines(IL-17A, IL-17F, IL-6, IL-22, and TNF-α) correlated with T17/Treg balance,
Nanoparticle curcumin	DSS -induced colitis mice	attenuated body weight loss, disease activity index, histological colitis score and improved mucosal permeability.	increased the relative abundance of Clostridium cluster IV and Clostridium subcluster XIVa, The fecal butyrate level significantly increased	increased expansion of CD4+ Foxp3+ regulatory T cells and CD103+ CD8α− regulatory dendritic cells

## Application of FMT

The ancient Chinese physician Ge Hong pioneered the use of fecal suspensions for the treatment of severe diarrhea (Drew, 2016). Manipulating the gut microbiota and its interaction with the intestinal immune system presents a safer and more sustainable approach to ameliorating patient symptoms. Nowadays, an increasing number of clinicians are employing standardized FMT techniques for the treatment of UC. A meta-analysis encompassing 31 studies on FMT for IBD demonstrated final remission rates of 39.6% for UC and 47.5% for CD, with an overall adverse event rate of less than 1% (Lai et al., 2019). A study conducted in China refined the process of extracting fecal bacteria and demonstrated, through *in vitro* and animal experiments, that washed microbiota transplantation is superior to manually prepared FMT. The washing process removes certain viruses and pro-inflammatory metabolites, thereby enhancing safety and the precision of microbial quantification (Zhang et al., 2020). Currently, an internationally standardized stool bank is lacking, and screening criteria for FMT donors vary. When the administration route of FMT proceeds through the lower digestive tract and the frequency of FMT administration increases, the efficacy of UC treatment significantly improves (Thangaraju et al., 2009). Therefore, optimizing FMT strategies, including administration routes, frequency, and dosage, will substantially enhance the treatment of UC. FMT addresses the dysbiosis observed in UC patients by introducing normal gut flora from healthy donors, aiding in the restoration of microbial balance within the gut. A study revealed that patients receiving FMT experienced an increase in microbial diversity but still exhibited lower diversity compared to the donors. In both UC and CD patients, clinical responders to FMT exhibited greater increases in α-diversity compared to non-responders. Post-transplantation, the introduced healthy microbiota can produce metabolic byproducts such as SCFAs, which contribute to lowering the intestinal pH. This reduction in pH can enhance bacterial and hydrogen peroxide adhesion, thereby competitively inhibiting the adhesion and translocation of pathogens and promoting the proliferation of beneficial bacteria (Yang et al., 2014). An untargeted metabolomic analysis showed that SCFAs and secondary bile acid (SBA) levels were higher in patients who responded after FMT compared to patients who received placebo enemas (Chen et al., 2023). Huang et al. observed that following FMT treatment, there was a marked decrease in the serum levels of inflammatory cytokines, such as TNF-α, IFN-γ, IL-1β, IL-6, and IL-8. Notably, in responders to FMT, the levels of TNF-α and IL-6 were significantly reduced (Fonseca-Camarillo and Yamamoto-Furusho, 2015). Jacob et al. observed a significant reduction in Th1 (IFNγ+CD4+) cells (*p* = 0.02) in rectal biopsies of UC patients by the 4th week post-FMT, while there was no difference noted in mucosal Th17 (IL-17+CD4+) cells. In summary, FMT also exerts its therapeutic effects by suppressing inflammatory responses (Jacob et al., 2017).

Given the increasing clinical application of FMT, variations in outcomes are observed, attributable to differences in donor composition and administration methods. Future research must adhere to standardized protocols to minimize bias. Comprehensive analyses, comparing pre- and post-FMT conditions, responders versus non-responders, and allogeneic versus autologous FMT, will be crucial. Such studies will facilitate the refinement of targeted therapies aimed at modulating the gut microbiome and/or metabolome.

## Application of probiotics

With the continued advancement of technology, next-generation probiotics have attracted significant interest. The purification and application of probiotics can effectively improve patient disease states. A meta-analysis, encompassing 12 randomized controlled trials (RCTs) with a total of 886 patients with UC, evaluated the efficacy of probiotics in UC. In subgroup analyses, probiotics significantly reduced the UC Disease Activity Index (UCDAI) and Disease Activity Index (DAI) in patients with active UC, while probiotics containing *Bifidobacteria* significantly reduced disease activity in these patients (Asto et al., 2019). A study showed that *Lactobacillus reuteri* could stimulate intestinal epithelial cell proliferation by increasing the expression of R-spondins, thereby activating the Wnt and β-catenin signaling pathways. *L. reuteri* maintains the number of Lgr5+ cells and stimulates intestinal epithelial proliferation to repair epithelial damage and reduce pro-inflammatory cytokine secretion in the intestine and LPS concentration in serum (Wu et al., 2020). A novel probiotic, *Lactobacillus entericus (L. Intestinalis)*, exerts a protective effect on DSS-induced colitis in mice. *L. Intestinalis* reduces C/EBPα-driven production of serum amyloid A family (SAA)1 and SAA2 in the intestinal epithelium, thereby affecting Th17 cell differentiation (Wang et al., 2023). Besides, *A. muciniphila*, a next-generation probiotic, is significantly diminished in UC patients. Recent studies have shown that *A. muciniphila* in the murine gut induces T cell-dependent immunoglobulin G1 (IgG1) antibody production at steady state. *In vitro* studies have shown that *A. muciniphila* enhances intestinal epithelial integrity and repairs damaged intestinal mucosal barriers. This may be attributed to metabolites of *A. muciniphila*. Current evidence suggests that *A. muciniphila* maintains homeostasis of the intestinal epithelium and suppresses the immune response of intestinal epithelial cells by degrading host intestinal mucus into short-chain fatty acids (Derrien et al., 2011). Previous studies have shown that *A. muciniphila*, its outer membrane protein Amuc_1100, and extracellular vesicles from *A. muciniphila* all improve colitis symptoms in mice and maintain intestinal mucosal barrier integrity. The mechanism by which *A. muciniphila* relieves colitis may be related to the interaction of Amuc_1100 with Toll-like receptor 2 (Wang et al., 2020). Research has revealed diminished colonization of *L. johnsonii*, a potential anti-inflammatory bacterium, in mice with colitis. Oral supplementation with *L. johnsonii* alleviates colitis by specifically increasing the proportion of intestinal macrophages and the secretion of IL-10. *L. johnsonii* activates primary macrophages into CD206+ macrophages that release IL-10 via the TLR1/2-STAT3 pathway, thereby mitigating experimental colitis (Jia et al., 2022). A study conducted by Tamaki et al. in 2016 applied *Bifidobacterium longum 536 (B. longum 536)* to patients with mild-to-moderately active UC, which improved clinical symptoms, including the UC Disease Activity Index and the Rachmilewitz Endoscopic Index. However, further research is necessary to fully establish the efficacy and safety of *B. longum 536* for UC (Tamaki et al., 2016). Besides, a single-center, randomized, double-blind, placebo-controlled study investigated the role of a mixture of three strains—*Streptococcus faecalis T-110*, *Clostridium butyricum TO-A*, and *Bacillus mesentericus TO-A*—in preventing relapse in patients with UC who were in remission. The study’s findings indicated a significantly higher rate of relapse at 3 and 9 months in the probiotic treatment group; however, no difference was observed at 12 months (Yoshimatsu et al., 2015). A highly regarded probiotic compound is VSL#3, which consists of four strains of *Lactobacilli* and three strains of *Bifidobacteria*. Research using murine models has shown that this probiotic blend can inhibit the expression of NF-κB and TNF within the TLR4-NF-κB signaling pathway (Bibiloni et al., 2005; Derwa et al., 2017).

A variety of probiotics have demonstrated therapeutic efficacy in animal models of UC. These mechanisms include competing for adhesion sites and nutrients, maintaining the equilibrium of the normal gut microbiota, enhancing intestinal mucosal barrier function, promoting intestinal mucosal immune tolerance, modulating gut inflammatory responses, and inhibiting intestinal epithelial cell apoptosis. However, further clinical investigations are warranted to establish their efficacy and safety in clinical applications.

## Application of prebiotics

Concomitant with the application of probiotics, the concept of prebiotics has also gained increasing prominence. Prebiotics are defined as a class of non-digestible food ingredients that selectively stimulate the growth and/or activity of one or a limited number of beneficial bacteria in the gut (such as *Bifidobacteria* and *Lactobacilli*), thereby conferring positive health effects on the host (Kennedy et al., 2024). The primary function of prebiotics is to serve as a substrate, promoting the proliferation of indigenous probiotics in the gut, rather than directly supplying viable microorganisms. Common prebiotics include inulin and fructooligosaccharides (FOS), among others, and encompass other fermentable carbohydrates, polyphenols, and polyunsaturated fatty acids. A study has shown that patients with mild to moderate UC who were administered inulin enriched with FOS exhibited significant improvements in clinical activity indices, a reduction in fecal calprotectin, and an increase in SCFA production (Casellas et al., 2007; Valcheva et al., 2019). Isomaltooligosaccharides (IMO), similar to inulin and xylooligosaccharides, are dietary fibers that improve stool volume and fecal acidity. IMO is readily metabolized by *Bifidobacteria* and other microorganisms, such as *Lactobacillus acidophilus*, *Bacteroides vulgatus*, and *Bifidobacterium bifidum*. IMO promotes the proliferation of *Bifidobacteria* and *Lactobacilli*, leading to localized and systemic Th1-like immune responses and modulation of immune function (Guarino et al., 2020). Prebiotics in UC can promote the growth of beneficial microorganisms, which compete with pathogenic species and produce beneficial fermentation products, such as SCFAs. SCFAs possess immunomodulatory properties and can influence TLR-4 signaling and pro-inflammatory cytokine production (van der Beek et al., 2017). A clinical study utilized mesalazine, inulin enriched with FOS, and a placebo to treat patients with mild to moderate UC. The study demonstrated that oral administration of inulin enriched with FOS could reduce fecal calprotectin levels in UC patients, an indicator of intestinal inflammation. This reduction suggests a potential therapeutic benefit of prebiotic supplementation in managing the symptoms and inflammatory status of UC (Casellas et al., 2007).

## Application of herb medicine

Traditional Chinese Medicine (TCM) has a history of application spanning thousands of years and, in contemporary clinical practice, leverages its multi-target advantages to effectively treat various diseases. Although the efficacy of TCM has been acknowledged, the mechanisms through which TCM exerts its effects on the human body remain incompletely elucidated. The quantity and distribution of gut microbiota are significantly influenced by orally administered or enema-delivered Chinese herbal medicines. These herbs may exhibit prebiotic-like effects, modulating the number, distribution, and metabolic products of gut microbiota to promote their prebiotic functions, thereby contributing to disease therapy. Research indicates that several individual Chinese herbal compounds as well as compound formulas can improve the gut microbiota in UC, impacting the immune system and ultimately alleviating UC symptoms. A study showed that total flavonoids from *Sophora flavescens* could ameliorate DSS-induced colitis in mice, repair the mucosal barrier, reduce the expression of inflammatory mediators, increase the abundance of *A. muciniphila* in the gut, and promote the growth of beneficial bacteria (Bu et al., 2021). The mechanisms underlying these effects may be related to the regulation of NF-κB and MAPK signaling pathways (Zhang et al., 2019). A study has shown that *Astragalus membranaceus* (Huangqi) extract, acting as a prebiotic, can alleviate DSS-induced ulcerative colitis in mice through a gut microbiota-dependent mechanism by restoring SCFA production and modulating the balance between Th17 and Treg cells (Zhang et al., 2025). A study demonstrated that extracts from *Codonopsis pilosula* could alleviate enteritis symptoms in mice by modulating the gut microbiota. The addition of *Codonopsis* polysaccharides increased the abundance of three probiotic species: *Bifidobacteria*, *Lactobacillus*, and *A. muciniphila*, while inhibiting the proliferation of pathogenic bacteria. Furthermore, it was found that *Codonopsis* polysaccharides selectively enhanced the population of SCFA-producing bacteria, promoting SCFA production, thereby augmenting their systemic and local functions and alleviating malnutrition symptoms in enteritis-affected mice. Moreover, *Codonopsis* polysaccharides mitigated intestinal mucosal damage in mice by suppressing the expression of pro-inflammatory cytokines and enhancing the expression of anti-inflammatory cytokines (Jing et al., 2018).

Traditional Chinese medicines, such as *Lonicera japonica*, *Artemisia capillaris*, and *Eucommia ulmoides* leaves, are rich in chlorogenic acid. Acting as a prebiotic, chlorogenic acid improved body weight loss and DAI scores in DSS-treated mice. Chlorogenic acid was found to reverse the DSS-induced downregulation of mucin proteins, maintaining intestinal barrier integrity. Moreover, chlorogenic acid significantly reduced serum levels of IFN-γ, TNF-α, and IL-6 in mice, decreased infiltration by neutrophils, macrophages, and T cells, and exhibited anti-inflammatory effects on the colonic mucosa of DSS-treated mice. Regarding microbial composition, at the phylum level, chlorogenic acid notably reduced the *Firmicutes*-to-*Bacteroidetes* (F/B) ratio compared to DSS-treated mice. At the genus level, the abundance of *A. muciniphila* was significantly increased, possibly due to its potent ability to scavenge oxygen free radicals (Zhang et al., 2017). Recent research has demonstrated that celastrol, derived from the thunder god vine (*Tripterygium wilfordii*), can increase colon length and repair the epithelial barrier in mice with DSS-induced colitis. Celastrol (CSR) promotes the differentiation of Th1, Th17, and Tregs, enhances the expression of anti-inflammatory mediators, and inhibits inflammatory cytokines and their signaling pathways, thereby maintaining intestinal immune homeostasis. Notably, this immunomodulatory effect was abolished following antibiotic treatment, indicating its dependence on an intact gut microbiota. Further FMT experiments confirmed that the therapeutic effects of celastrol were microbiota-dependent. CSR treatment was found to alter the structure of the gut microbiota and its metabolic products, which in turn regulated immune responses to ultimately alleviate colitis (Li et al., 2022). *Pulsatilla* Decoction is a commonly used drug for treating diarrhea in ancient Chinese medicine. Three recent studies have shown that Pulsatilla Decoction can alleviate UC induced by DSS by regulating intestinal flora and the IL-6/STAT3 signaling pathway (Xuan-Qing et al., 2021), regulating the GM and BAs through FXR and TGR5 signaling pathway (Hua et al., 2021), improving Th17/Treg balance and intestinal epithelial barrier (Miao et al., 2020). *Pulsatilla chinensis* is the key Chinese medicine in *Pulsatilla* decoction. Studies have shown that *Pulsatilla chinensis* extract reduces DAI in a 3% DSS-induced ulcerative colitis mouse model. *Pulsatillae radix* extract was found to treat intestinal microbiota disorders manifested by reductions in *Bacteroides*, *Parabacteroides*, *Prevotella*, Faecalibacterium, *Spirochaeta*, and *Escherichia coli*. Treatment with *Pulsatillae radix* extract enhanced NOD-like receptor signaling while inhibiting cytokine-cytokine receptor interactions and TNF and IL-17 signaling pathways (Li et al., 2023). Kuijieyuan Decoction (KD) is a traditional Chinese medicine prescription that draws upon classical Chinese medical theories and texts. It has been developed to address the symptoms of UC and is used in clinical settings for this purpose. Treatment with KD reduced the DAI score in rats with UC and increased colon length. KD administration also enriched the abundance of *Alloprevotella*, *Treponema*, *Prevotellaceae*, and *Prevotella*. The mechanisms underlying these effects may involve the attenuation of the TLR4-dependent PI3K/AKT/NF-κB signaling pathway (Liu et al., 2020).

One study showed that rhubarb peony decoction relieved colitis symptoms in mice, which can promote the growth of *Firmicutes, Actinobacteria, Butyricoccus, Pullicaecorum* and *Lactobacillus*, increase the content of SCFAs. The mechanism may be related to restore the Th17/Treg homeostasis. Another study showed that Codonopsis pilosula Nannf (CPN) polysaccharides also regulate intestinal flora, increasing the content of acetic acid, propionic acid, isobutyric acid, butyric acid and isovaleric acid, which regulates the expression of proinflammatory and anti-inflammatory cytokines associated with the Th17/Treg balance (Jing et al., 2018). A study showed that Flos Abelmoschus manihot extract (AM) significantly alleviated DSS-induced colitis in mice. AM elevated the abundance of SCFAs-producing gut microbiota in colitis mice. Consequently, levels of SCFAs especially butyrate and acetate were increased upon AM treatment, which, primarily through peroxisome proliferator-activated receptor gamma (PPARγ) pathway, led to the enhanced Treg generation and the suppressed Th17 development (Zhang et al., 2019). Recent research examined the effects of nanoparticle curcumin (named Theracurmin) on experimental colitis in mice. Treatment with nanoparticle curcumin significantly attenuated body weight loss, disease activity index, histological colitis score. Treatment with nanoparticle curcumin increased the abundance of butyrate-producing bacteria and fecal butyrate level. This was accompanied by increased expansion of CD4+ Foxp3+ regulatory T cells and CD103+ CD8α− regulatory dendritic cells in the colonic mucosa. Treatment with nanoparticle curcumin suppressed the development of DSS-induced colitis potentially via modulation of gut microbial structure. These responses were associated with induction of mucosal immune cells with regulatory properties (Ohno et al., 2017).

## Conclusion and prospects

The pathogenesis of UC is multifactorial, and a comprehensive understanding of its mechanisms provides novel insights into the search for potential therapeutic interventions and the enhancement of existing treatments. In recent years, a growing body of research has elucidated the significant role that the GM plays in the pathogenesis of UC. Gut microbiota dysbiosis and alterations in its metabolites can lead to intestinal immune responses through various pathways, thereby initiating and promoting the development of UC. A variety of therapeutic modalities targeting this potential mechanism have emerged, including FMT, which aims to improve patient conditions by reconstructing a healthy gut microbial community. In addition to FMT, probiotics, prebiotics, and TCM have also been extensively studied as treatment options for UC. Traditional probiotics, such as *Lactobacillus* species, and next-generation probiotics like *A. muciniphila* have been shown to modulate gut immunity by altering the profile of gut metabolites, thereby ameliorating symptoms of DSS-induced colitis in mice. Prebiotics and TCM are similarly widely utilized in the treatment of UC, alleviating UC-related gut dysbiosis by augmenting beneficial bacterial populations, diminishing potentially pathogenic bacterial populations, and/or modulating microbially mediated metabolites. Advancements in multi-omics technologies, such as genomics, proteomics, and metabolomics, will offer novel perspectives on the in-depth understanding of UC pathogenesis. Considering the potential for personalized medicine, variations in microbial composition among individuals exist. Moreover, as our understanding of the interaction between gut microbiota and the host deepens, more innovative therapies targeting gut barrier function and immune modulation mechanisms may emerge. Finally, given the impact of antibiotic utilization on the gut microbiome, judicious prescribing will also be a critical aspect of optimizing UC therapy.

In conclusion, future research should persist in exploring these areas to advance our knowledge and develop innovative strategies for the management and treatment of UC.
